# Congenital dacryocystocele diagnosed by antenatal ultrasonography with spontaneous resolution

**DOI:** 10.5935/0004-2749.20200055

**Published:** 2020

**Authors:** Gheorghe Cruciat, Andreea Florian, Paul Cotutiu, Georgiana Nemeti, Simona Nicoara

**Affiliations:** 1 Department of Obstetrics and Gynecology, “Iuliu Hatieganu” University of Medicine and Pharmacy, Cluj-Napoca, Romania; 2 Department of Ophthalmology, “Iuliu Hatieganu” University of Medicine and Pharmacy, Cluj-Napoca, Romania

**Keywords:** Dacryocystitis/congenital, Dacryocystitis/diagnostic imaging, Lacrimal duct obstruction/congenital, Lacrimal duct obstruction/diagnostic imaging, Ultrasonography, prenatal, Dacriocistite/congênito, Dacriocistite/diagnóstico por imagem, Obstrução dos ductos lacrimais/congênito, Obstru ção dos ductos lacrimais/diagnóstico por imagem, Ultranosografia pré-natal

## Abstract

Dacryocystocele is a rare benign facial abnormality of the nasolacrimal system, which may be detected at the antenatal workup during the third trimester of pregnancy. Ultrasound is the method of choice for this examination. However, magnetic resonance imaging may also be used in selected cases. Dacryocystocele is mostly a transient finding; it may resolve spontaneously in utero or postnatally. When the defect is bilateral and persists in neonatal life, it may lead to respiratory complications. We report a case of a fetus with bilateral dacryocystocele diagnosed by prenatal ultrasound at the beginning of the third trimester of pregnancy with spontaneous postpartum resorption.

## Abstract (pt)

A dacriocistocele é uma anormalidade facial benigna rara do sistema nasolacrimal, que pode ser detectada na rotina pré-natal durante o terceiro trimestre da gravidez. O ultrassom é o método de escolha, mas a ressonância magnética também pode ser usada em casos específicos. Na maioria das vezes, a dacriocistocele é um achado temporário, que pode se resolver espontâneamente ainda no útero ou após o nascimento. Quando a anormalidade é bilateral e persiste na vida neonatal, pode levar a complicações respiratórias. Este é o relato do caso de um feto com dacriocistocele bilateral diagnosticada por ultrassom pré-natal no início do terceiro trimestre da gravidez, com reab sorção espontânea após o nascimento.

## INTRODUCTION

Congenital dacryocystocele (DCC) is an uncommon variant of nasolacrimal duct obstructions, with an incidence of 0.1% and potential spontaneous postpartum resolution^(1-3)^. Detection of this condition through prenatal ultrasound during the third trimester of pregnancy has been reported in the literature; however, only a few of these cases were bilateral^(1)^.

We present a case of bilateral DCC diagnosed by prenatal ultrasound at 31 weeks of gestation (WG) that res olved spontaneously after birth, with normal successive postnatal follow-ups.

## CASE REPORT

A 40-year-old pregnant patient, gravida II, para II, with a prior delivery through Cesarean section due to fetal macrosomy and maternal-fetal disproportion, presented for routine prenatal ultrasound scanning scheduled at 31 WG. The evolution of pregnancy was physiologic until presentation, with a normal fetal karyotype (46XX), as indicated by amniocentesis performed due to maternal age. Second trimester morphology was normal.

**Figure 1:**
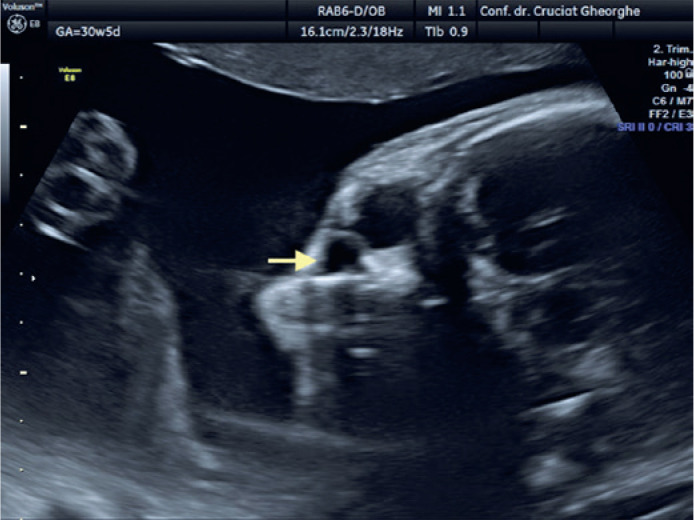
Dacryocystocele on the right side at 31 weeks of gestation (arrow).

**Figure 2:**
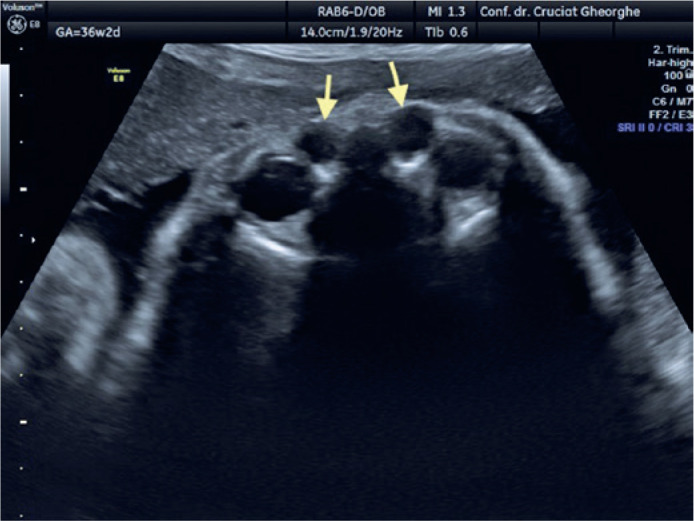
Bilateral dacryocystocele at 36 weeks of gestation (arrows).

**Figure 3:**
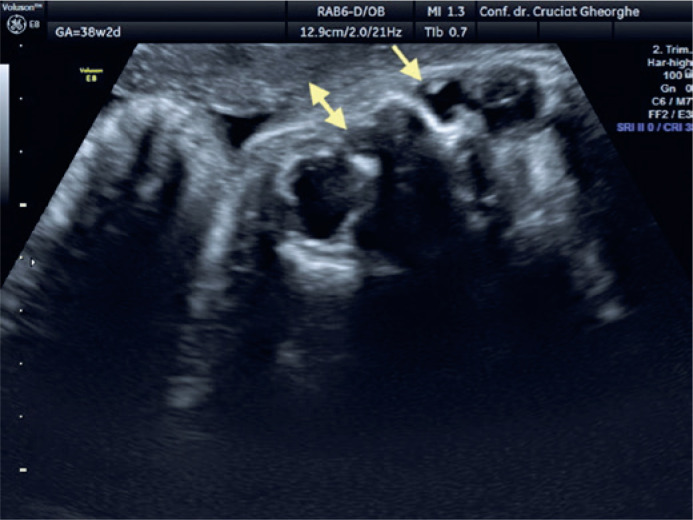
Bilateral dacryocystocele at 38 weeks of gestation with persistence of congenital dacryocystocele (arrow) on the right side and progressive resolution of the cyst on the left side (double arrow).

Examination of the fetal cephalic pole using fetal ultrasound performed at 31 WG revealed the presence of a 3-mm, liquid-filled, hypoechoic cyst located inferiorly and separated from the right eye ball (Figure 1). At 33 WG, the cystic lesion had enlarged (6/4.5 mm); however, it remained well demarcated from the eye ball and the nasal fossa. At the 36 WG follow-up examination, the dimensions of the right cyst were 8/7 mm and a similar lesion of 6/4.5 mm was identified contralaterally (Figure 2). At 38 WG, the DCC on the left side diminished and its content became more dense/opaque, whereas the structure on the right side maintained its liquid content and increased in size to 8/8.5 mm (Figure 3). All ultrasound examinations were performed using a Voluson E8 ultrasound system (GE Medical Systems, Zipf, Austria). Elective Cesarean section was performed at 39 WG, without perioperative complications. The newborn weighed 3,800 g, with an Apgar score of 10.

### Postpartum evolution

Postpartum ophthalmological examination of the newborn revealed a bluish, cystic swelling, 15 mm vertical/12 mm horizontal in size, located immediately below the right medial canthal tendon, with a firm consistency. Superior and inferior puncta lacrimalia were normal. The lacrimal system appeared normal on the left side. There was no abnormality in the anterior and posterior segments of the eyes. Pupils were reactive to light and pupillary reflexes were normal. The Hirshberg test demonstrated normally aligned eyes. Clinical features were consistent with the diagnosis of congenital DCC.

### Treatment

Oral antibioprophylaxis with amoxicillin/clavulanate 500/125 mg once every 12 h was initiated to reduce the risk of infectious complications (dacryocystitis). At day 1 following treatment, the mother reported a significant discharge from the medial canthal area, followed by spontaneous decompression of the bluish cyst. Topical treatment with a combination of netilmicine sulfate (3 mg/ml) and dexamethasone disodium phosphate (1 mg/ml) four times daily for 5 days was prescribed, followed by complete resolution of the DCC without sequelae. Nasal examination was performed 1 week postnatally to rule out coexistent nasal cysts and the complete bilateral resolution of the DCC was confirmed. Nasal endoscopy and ophthalmological examinations at 1-, 6-, and 12-month follow-ups were normal.

## DISCUSSION

Antepartum diagnosis of bilateral DCC is scarcely reported, probably due to their spontaneous resorption, at least homolaterally. In the present case, detection of bilateral DCC at 31 WG was followed by complete antenatal resorption of the cyst unilaterally and postnatal resolution of the contralateral DCC with conservative treatment and antibioprophylaxis.

During development, the lacrimal drainage system in embryos has been described as early as 5 weeks. By week 10, a lumen is formed in the lacrimal cord; canalization of this lumen results in the connection and communication with the inferior meatus, which is completed between the 26 WG and beyond term^(2)^. Anatomic studies showed that the lower end of the duct remains covered by the Hasner membrane (i.e., the apposed mucosal linings of the lower ductal end and the nasal fossa) in 35-73% of full-term fetuses; this resolves spontaneously in 85%95% of cases during the first year after birth^(2)^.

DCC is caused by obstruction of the nasolacrimal system at two sites: distally (Hasner valve) and proximally (Rosenmuller valve). The first obstruction is anatomical and the second one is functional, being caused by the distended sac that functions as a trap-door^(2)^. The sac becomes filled either with mucus (mucocele) or amnion (amniocele). Most cases of DCC resolve spontaneously in utero or immediately after birth (91% prior to the sixth month of life)^(1)^. In case of persistent DCC, secondary dacryocystitis may develop within days or weeks^(3)^.

The literature shows predominance of unilateral lesions (75%)^(1,2)^. DCC is more common in females because the lacrimal duct is constitutionally narrower^(1,4)^.

Ultrasound is the method of choice for fetal abnormality screening owing to its superior spatial resolution, availability, cost, and lack of exposure to radiation. Most cases of DCC reported in the literature were identified during routine ultrasound scans performed in the third trimester (after 30 WG), such as in the present case^(5-8)^. Hemangioma was excluded from the differential diagnosis due to its typical late occurrence postpartum and softer consistency. Encephalocele, nasal glioma, and dermoid cyst were ruled out because they are located superiorly to the medial canthal tendon.

Magnetic resonance imaging examination may diagnose DCC earlier in gestation and detect smaller lesions. However, it is only used when associated malformations are suspected or differential diagnosis with encephalocele is sought^(9)^. Magnetic resonance imaging detection leads to a peak WG at diagnosis of 27 weeks, with a progressive decrease in incidence toward term. This is probably attributed to cases which achieve complete perforation of the nasolacrimal duct followed by cyst resorption^(9)^.

DCC may appear isolated or in association with other ab normalities or syndromes, such as ectrodactyly-ectrodermal dysplasia clefting, Down syndrome, Canavan disease, pyelectasis, and dysplastic kidney; rendering early diagnosis crucial^(5,10)^.

Although isolated DCC is a benign condition, the provision of counseling is mandatory to reduce parental anxiety and optimize perinatal care.
